# Elucidating the impact of biochar with different carbon/nitrogen ratios on soil biochemical properties and rhizosphere bacterial communities of flue-cured tobacco plants

**DOI:** 10.3389/fpls.2023.1250669

**Published:** 2023-09-15

**Authors:** Yingfen Yang, Chenghu Ye, Wei Zhang, Xiaohong Zhu, Haohao Li, Dehai Yang, Waqar Ahmed, Zhengxiong Zhao

**Affiliations:** ^1^Yunnan Agricultural University, Kunming, Yunnan, China; ^2^Yunnan Revert Medical and Biotechnology Co., Ltd., Kunming, Yunnan, China; ^3^Kunming Branch of Yunnan Tobacco Company, Kunming, Yunnan, China; ^4^Hongta Tobacco Group Limited Company, Dali, Yunnan, China

**Keywords:** biochar, soil biochemical properties, nitrogen fertilizer, rhizosphere bacterial communities, biomass accumulation

## Abstract

**Background and aims:**

In agriculture, biochar (BC) and nitrogen (N) fertilizers are commonly used for improving soil fertility and crop productivity. However, it remains unclear how different levels of BC and N fertilizer affect soil fertility and crop productivity.

**Methods:**

This study elucidates the impact of different application rates of BC (0, 600, and 1200 kg/ha) and N fertilizer (105 and 126 kg/ha) on biomass accumulation, soil microbial biomass of carbon (SMC) and nitrogen (SMN), and soil biochemical properties, including soil organic carbon (SOC), total nitrogen (TN), soil nitrate nitrogen (NO_3_^−^−N), ammonium nitrogen (NH_4_^+^−N), urease (UE), acid phosphatase (ACP), catalase (CAT), and sucrase (SC) of tobacco plants. In addition, a high throughput amplicon sequencing technique was adopted to investigate the effect of different application rates of BC/N on rhizosphere bacterial communities of tobacco plants.

**Results:**

The results confirm that high dosages of BC and N fertilizer (B1200N126) significantly enhance dry matter accumulation by 31.56% and 23.97% compared with control B0N105 and B0N126 under field conditions and 23.94% and 24.52% under pot experiment, respectively. The soil biochemical properties, SMC, and SMN significantly improved under the high application rate of BC and N fertilizer (B1200N126), while it negatively influenced the soil carbon/nitrogen ratio. Analysis of rhizosphere bacteriome through amplicon sequencing of 16S rRNA revealed that the structure, diversity, and composition of rhizosphere bacterial communities dramatically changed under different BC/N ratios. Proteobacteria, Bacteroidetes, Actinobacteria, Firmicutes, and Acidobacteria were highly abundant bacterial phyla in the rhizosphere of tobacco plants under different treatments. Co-occurrence network analysis displayed fewer negative correlations among rhizosphere bacterial communities under high dosages of biochar and nitrogen (B1200N126) than other treatments, which showed less competition for resources among microbes. In addition, a redundancy analysis further proved a significant positive correlation among SMC, SMN, soil biochemical properties, and high dosage of biochar and nitrogen (B1200N126).

**Conclusions:**

Thus, we conclude that a high dosage of BC (1200 kg/ha) under a high application rate of N fertilizer (126 kg/ha) enhances the biomass accumulation of tobacco plants by improving the soil biochemical properties and activities of rhizosphere bacterial communities.

## 1 Introduction

Biochar, which is produced through the incomplete combustion or geothermal carbonization of diverse biomass, such as animal manures, sewage sludge, and woody biomass, has beneficial impact on plant growth, soil enrichment, and climate change (Chen et al., 2021). Generally, pyrolysis and hydrothermal carbonization (HTC) techniques are used for biochar production. Pyrolysis is a conventional process which thermally transforms biomass into a solid product (pyrochar) under anaerobic conditions and at moderate temperature (350-700°C) (Akhtar et al., 2018; Li et al., 2022). HTC, however, transforms biomass into a solid product (hydrochar) utilizing water as the reaction medium at considerably gentler temperature (130-250°C) and self-generated pressure (2-6 MPa) (Qin et al., 2017). The various HTC and pyrolysis processes endow hydrochar and pyrochar with distinct properties (e.g., surface area, element content, and surface functionalization). For example, biochar contains a huge quantity of micro-/macro-nutrients and carbon contents (70−80%) and a large surface area, while hydrochar contains higher oxygen contents (Liu et al., 2010; Ronsse et al., 2013; Wiedner et al., 2013).

In recent years, biochar application has been gaining much attention of researchers due to its potential role in waste mitigation, lessening of greenhouse gas emissions, carbon sequestration, ecological restoration, soil amendment, and disease suppression (Lehmann and Joseph, 2015; Li et al., 2022; Wang et al., 2022). Soil amendments with biochar increase soil fertility and crop productivity under different agroecosystems by improving soil organic matter contents, nutrient retention, acquisition ability, and plant growth despite repeated cultivations (Pandey et al., 2016; Khan et al., 2021a). Various agricultural waste materials are used to produce diverse types of biochar that modulate soil health and physicochemical properties (Streubel et al., 2011). For example, regarding improvement in soil nutrients, cedar leaf biochar was shown to be superior to sawdust biochar but reduced the soil’s total carbon contents (Hu et al., 2019). However, a high dosage of biochar negatively influences soil fertility, plant growth and yield, short-term reduction in the availability of soil mineral nutrients, and soil microbial activities (Tammeorg et al., 2014; Khan et al., 2022).

Thus, an appropriate biochar application rate is essential for increase crop yield, soil fertility, and soil adsorption capacity (Peng et al., 2021). For instance, it is reported that biochar application at a certain range (1−4%) improves soil fertility, nutrient uptake ability of plants, and crop yield. While application of biochar >5% reduces soil fertility and inhibits plant growth (Pokovai et al., 2020; Zheng et al., 2020). Excessive biochar applications (more than 12 t ha^−1^) negatively influenced chlorophyll contents, the photosynthesis rate of maize crops, and soil total nitrogen contents (Khan et al., 2023). Furthermore, the application of biochar improves the systemic resistance of plants against a variety of plant pathogens, such as in strawberries, against *Colletotrichum acutatum* and *Botrytis cinerea* (Meller Harel et al., 2012) and in tobacco, against *Ralstonia solanacearum*, the causative agent of tobacco bacterial wilt (Li et al., 2022). However, the efficacy of biochar in disease suppression is varied according to biochar type, dosage, nature of phytopathogen, and host (Graber et al., 2014; De Tender et al., 2016).

The plant rhizosphere is one of the most intricate ecosystems on the planet and a hotspot habitat for various microorganisms (Raaijmakers, 2015). The addition of biochar has a substantial effect on the diversity and structure of rhizosphere microorganisms (Chen et al., 2013), such as *Pseudomonas*, *Bacillus*, and *Lysobacter* Spp., which play a vital role in the decomposition of organic matter, nutrients cycle, maintaining soil carbon/nitrogen (C/N) ratio, nitrogen fixation, and mineralization of soil nutrients (Fuke et al., 2021). Thus, the effectiveness of rhizosphere microbial diversity and soil nutrients are the key factors for healthy plant growth (Ren et al., 2022). Previous studies have reported that soil amendment with biochar can change the soil physicochemical properties and structure and the composition of rhizosphere microbial communities (Prayogo et al., 2014; Xu et al., 2014). Therefore, it is suggested that applying biochar at a specific range is essential for improving soil health, diversity of rhizosphere microbial community, and metabolic activities of rhizospheric microorganisms (Ren et al., 2022).

For instance, the soil C/N ratio and microbial biomass carbon significantly increased under biochar dose < 40 t/hm^2^, while application of biochar > 40 t/hm^2^ decreased the functional diversity, metabolic activity, and richness indexes of rhizosphere microorganisms (Zhou and Chen-yang, 2019). Nitrogen (N) is a critical macronutrient for plant growth, development, and high yield (Song et al., 2022). The unnecessary use of N fertilizers limits economic benefits and cause environmental hazards, such as water contamination, air pollution, and soil acidification (Xia et al., 2020). It has been reported that soil amendment with biochar improves plants’ nitrogen use efficiency and reduced nitrogen loss, thus enhanced plant growth and yield (Seyedghasemi et al., 2021; Wang et al., 2023). Therefore, the combined application of biochar and N fertilizer is recommended to mitigate the environmental hazards caused by N fertilizer and to improve plants’ nitrogen use efficiency (Xia et al., 2022). However, the impact of the high application rate of biochar and N fertilizer on crop productivity, soil biochemical properties, and rhizosphere bacterial communities remains unclear.

Tobacco is an important industrial crop in China, broadly cultivated in the south-central regions of China (Ahmed et al., 2022). Thus, farmers use a massive volume of N fertilizers yearly to obtain a high yield of flue-cured tobacco leaves. In addition, tobacco stems that comprise 25–30% of the weight of the tobacco leaves are left over after leaf collection. Nowadays, burning tobacco stems is the most common approach, which causes environmental pollution and wastage of natural resources. In this study, tobacco stem biochar was used as a soil amendment in pot and field experiments to develop a system for the better utilization of natural resources. The objective of this study is to address several important questions regarding the optimal use of biochar and N fertilizer in tobacco crops. Specifically, the study aims to investigate how various combinations of biochar and N fertilizer influence soil biochemical properties, biomass accumulation, and rhizosphere bacterial communities of tobacco plants. We hypothesized that different application rates of C/N ratio would have a distinct influence on soil enzymatic activities, soil nutrients, and rhizosphere bacterial community compared with non-treated, which may affect tobacco plants’ growth and biomass accumulation. Overall, this study provides experimental-based knowledge to better utilize agricultural resources for the high-yield and quality production of tobacco leaves.

## 2 Materials and methods

### 2.1 Experimental site and material description

Pot and field experiments were conducted during the two growing seasons from April to September in 2021 and April to September in 2022 in Kunming City (25° 02′ 11″ N, 102° 42′ 31″ E) and Malong County, Qujing City (25° 29′ 27.6″ N, 103° 47′ 45.6″ E) Yunnan Province, China, respectively. The greenhouse conditions were maintained at a 28°C day temperature and a 22°C night temperature, with 14 h light and 10 h dark photoperiods. At the field conditions, the climatic conditions were recorded as follows: annual rainfall of 927.1 mm, average yearly temperature of 14.3°C, and 2158 sunshine hours per year with an average of 234 frost-free days. Tobacco stem biochar was used as a soil amendment, prepared by pyrolysis at 450°C from Kunming Canghui Co. Ltd. Yunnan, China, and passed through a 3 mm sieve before application. The basic physicochemical properties of biochar and soil used in these experiments are listed in **Tables S1**, **S2**.

### 2.2 Pot experiment

The pot experiment was conducted using the flue-cured tobacco cultivar “Yun87” in the greenhouse from April to September 2021 in Kunming City, Yunnan. The experiment was performed under different carbon/nitrogen (C/N) ratios according to 0, 600, and 1200 kg/ha biochar and 0, 105, and 126 kg/ha pure nitrogen. The experiment was performed under six different treatments as follows: 0 g/pot of biochar + 7 g/pot of pure nitrogen (B0N105), 40 g/pot of biochar + 7 g/pot of pure nitrogen (B600N105), 80 g/pot of biochar + 7 g/pot of pure nitrogen (B1200N105), 0 g/pot of biochar + 8.4 g/pot of pure nitrogen (B0N126), 40 g/pot of biochar + 8.4 g/pot of pure nitrogen (B600N126), and 80 g/pot of biochar + 8.4 g/pot of pure nitrogen (B1200N126). Seedlings (50 days old) of tobacco cultivar “Yun87” were transferred into pots (40 × 37 cm) comprising 20 kg of paddy soil and biochar dosage and irrigated three times a week (1000 mL/pot). To overcome the nutrient shortage, fertilizer was applied as a base and top fertilizer (70:30) in the form of tobacco-specific compound fertilizer (N: P_2_O_5_: K_2_O = 10: 10: 20) as a 1:1:3 ratio (Cai et al., 2021). Whole biochar and 70% of the base fertilizer were applied before transplanting the seedlings, while 30% of the top fertilizer was applied at 25 days of post-transplantation. The experiment was performed three times using 180 plants (30 plants per treatment and 10 plants per replicate) under a complete block design.

### 2.3 Field experiment

The field experiment was conducted using the flue-cured tobacco cultivar “Yun121” from April to September 2022 in Qujing City, Yunnan. Tobacco field preparations such as ridge raising, biochar amendment, and farm fertilizer applications were conducted in April 2022. The experiment was conducted under different C/N ratios of 0, 600, and 1200 kg/ha biochar and 105 and 126 kg/ha pure nitrogen. Tobacco-specific compound fertilizer (N: P_2_O_5_: K_2_O) was used as a 1:1:3 ratio in the form of base and top fertilizer (70:30) (Cai et al., 2021). After the ridge preparations, holes were made on the ridges with plant × plant (60×60 cm) and row × row (120×120 cm) distance, and whole biochar and 70% of the base fertilizer were applied in the holes and mixed thoroughly with the soil. Tobacco seedlings (50 days old) of the cultivar “Yun121” were transplanted on the ridges in the holes and the experiment was executed under six different conditions as follows: 0 kg/ha of biochar + 105 kg/ha of pure nitrogen (B0N105), 600 kg/ha of biochar + 105 kg/ha of pure nitrogen (B600N105), 1200 kg/ha of biochar + 105 kg/ha of pure nitrogen (B1200N105), 0 kg/ha of biochar + 126 kg/ha of pure nitrogen (B0N126), 600 kg/ha of biochar + 126 kg/ha of pure nitrogen (B600N126), and 1200 kg/ha of biochar + 126 kg/ha of pure nitrogen (B1200N126). The remaining 30% of the top fertilizer was applied with irrigation water at 25 days of post-transplantation. The integrated field management approaches were adopted according to China’s National Standards of the Tobacco Industry (Cai et al., 2021). The experiment was repeated three times under a randomized complete block design with 18 plots (3 plots per treatment), each containing 80 tobacco plants.

### 2.4 Samples collection and analysis of different indexes

To assess biomass accumulation in different plant parts (leaf, stem, and root) and soil biochemical properties, tobacco plant and soil samples were collected in replicates from pot and field experiments under different treatments after 85 (at the early baking stage) days post-transplantation. Briefly, three cores of plant and soil samples from the pot experiment/replicate and five cores from the field experiment/plot were taken and mixed thoroughly to make one composite sample per replication.

#### 2.4.1 Assessment of soil biochemical properties

Bulk soil samples were collected at a depth of 10-20 cm with a shovel (5 cm in diameter) by following the zig-zag sampling method and sieved through a 2-mm mesh and air-dried naturally to analyze soil physicochemical properties and enzymatic activity. The contents of soil organic carbon (SOC; g/kg) and total nitrogen (TN; g/kg) were determined by using the K_2_Cr_2_O_7_ oxidation external heating method and elemental analyzer (Elementar Analysensysteme GmbH, Germany), respectively (Khan et al., 2021b). Soil nitrate-nitrogen (NO_3_^−^−N; mg/kg) and ammonium nitrogen (NH_4_^+^−N; mg/kg) were extracted with indophenol-blue colorimetric and 1 M KCl methods, and their concentrations were measured at OD_275_ and OD_220_ nm using a spectrophotometer (UV-6000, China), respectively (Singh and Mavi, 2018). The chloroform fumigation method was used to determine the amount of soil microbial biomass of carbon (SMC) and soil microbial biomass of nitrogen (SMN). The activity of soil urease (S-UE) was determined by the indophenol blue colorimetric method. Whereas the activities of soil acid phosphatase (S-ACP), soil catalase (S-CAT), and soil sucrase (S-SC) were determined by the colorimetric method (Guan et al., 2022).

#### 2.4.2 Determination of biomass accumulation

The biomass accumulation in different parts of tobacco plants was determined through incubation (Li et al., 2022). Tobacco plant parts, including leaf, stem, and root, were incubated for 30 min at 105 °C and immediately dried for 48 h at 80 °C. The biomass accumulation in each part (leaf, stem, and root) was recorded to calculate whole plant biomass accumulation (g/plant).

Whole plant biomass accumulation (g/plant) = ∑ (biomass accumulation in leaves + stems + roots)

### 2.5 Investigation of rhizosphere bacterial community composition

#### 2.5.1 Rhizosphere soil samples collection and DNA extraction

In total, 18 rhizosphere soil samples (three samples/treatment) were collected to analyze rhizosphere bacterial community composition from the flue-cured tobacco plants at 85 days of post-transplantation from the field experiment, as described by Zhang et al. (2022). Five plants/plot from each treatment were uprooted, and soil particles attached to the root surface were collected using a brush and mixed to make one composite sample. According to the manufacturer’s instructions, 0.5 g of soil per sample was used to extract soil DNA using HiPure^®^ Soil DNA Kits (Magen, Guangzhou, China). The extracted DNA was stored at −80°C to analyze rhizosphere bacterial community composition and diversity.

#### 2.5.2 PCR amplification and library preparation

The V3-V4 region of the 16S gene of bacteria was amplified using primer pair 341F (5´- CCTAYGGGRBGCASCAG -3´) and 806R (5´- GGACTACNNGGGTATCTAAT-3´) (Ahmed et al., 2022) and sequenced on an Illumina MiSeq platform at Genedenovo Biotechnology Co., Ltd (Guangzhou, China). Raw data obtained from 16S *rRNA* amplicon sequencing were processed through Trimmomatic software (Version 0.33) and UCHIME (Version 8.1) for quality control (score *<* 20) and Chimera’s removal, respectively, to generate clean reads (Edgar et al., 2011; Bolger et al., 2014). The obtained clean reads were clustered into operational taxonomic units (OTUs) at a 97% similarity level using the UPARSE pipeline (Edgar, 2013). The taxonomic annotation of bacterial OTUs was done at a 70% threshold level using the SILVA database (Quast et al., 2012).

#### 2.5.3 Bioinformatics analysis

Quantitative insights in the microbial ecology pipeline (QIIME 2) were used to compute alpha diversity metrics and beta diversity based on Bray–Curti’s dissimilarity matrix for bacterial communities. The results of alpha and beta diversity metrics were visualized by boxplots and principal coordinate analysis (PCoA) using the R packages “ggplot2” and “Vegan”, respectively (Ahmed et al., 2022). Permutational multivariate analysis of variance (PERMANOVA) was performed according to “Adonis” using the vegan package in R (v.4.2.1). The interaction between common and unique OTUs within a group was visualized by “UpSet plots” in the UpSetR package in R (v.4.2.1). A Venn diagram was used to calculate the common and unique OTUs among the treatments and the result was visualized by “ggplot2” in R (v.4.2.1). The relative abundance (RA) of most abundant bacterial phylum, and genus was determined based on the OTU classified reads and plotted using R scripts in R package “ggplot2”. Redundancy analysis (RDA) was performed in the “Vegan package” in R (v.4.2.1) under different application rates of C/N ratio to confirm the relationship between rhizosphere bacterial communities, soil physicochemical properties and enzymatic activities, SMC, and SMN. Network analysis was performed at the phylum level within phylum (*p* < 0.05 and correlation coefficient > 0.9) using the “sparcc package” and visualized by the “psych package” in R (v.4.2.1).

### 2.6 Statistical analysis

The analysis of variance (ANOVA) in IBM SPSS V.20.0 (SPSS Inc., USA) was used to statistically assess the data about soil physicochemical parameters, enzymatic activities, biomass accumulation, and bacterial diversity in the rhizosphere. The results are shown as the standard error of means ( ± SEM), and the least significant difference (LSD) test was performed to identify differences among groups and was considered significant at *p* < 0.05. All figures were processed and combined using Adobe Illustrator 2019.

## 3 Results

### 3.1 Impact of varying C/N ratios on contents of SOC, TN, C/N ratio, NO_3_^−^−N, and NH_4_^+^−N

The levels of soil organic carbon (SOC), total nitrogen (TN), carbon/nitrogen (C/N) ratio, and nitrate nitrogen (NO_3_^−^−N) of flue-cured tobacco plants significantly changed (LSD; *p* < 0.05) with the increased application rate of biochar (0, 600, and 1200 kg/ha) under two nitrogen levels (105 and 126 kg/ha) in pot and field experiments, while no significant difference (LSD; *p* > 0.05) was observed for the contents of ammonium nitrogen (NH_4_^+^−N) among the treatments (**Table 1**). The level of SOC increased with the increased application rates of the C/N ratio in both the pot and field experiments. The contents of SOC were found to be significantly higher under treatment B1200N105 (30.04 g/kg) and B1200N126 (29.11 g/kg) in the pot experiment and under treatments B1200N105 (24.07 g/kg) and B1200N126 (23.57 g/kg) in the field experiment compared with B0N105 and B0N126 treatments and increased by 63.53%, 62.72%, 68.44%, and 69.32%, respectively (LSD, *p* < 0.05; **Table 1**). The contents of soil TN increased with the increased application rate of carbon and nitrogen and were found to be significantly higher under treatment B1200N126 (2.43 and 1.58 g/kg) in both the pot and field experiments, respectively, compared to other treatments (LSD, *p* < 0.05; **Table 1**). In both experiments (pot and field), the soil C/N ratio of flue-cured tobacco plants increased with the increased biochar and nitrogen fertilizer application rate. However, the soil C/N ratio of flue-cured tobacco plants was found to be significantly higher under treatment B1200N105 in the pot (13.39) and field (17.44) experiments compared with other treatments (LSD, *p* < 0.05; **Table 1**). In both experiments (pot and field), contents of NO_3_^−^−N first increased then decreased with the increased application rate of biochar under 105 kg/ha application of nitrogen fertilizer and were found to be significantly higher under treatment B600N105 (29.82 and 18.27 mg/kg) than B0N105 (LSD, *p* < 0.05; **Table 1**). Whereas, under 126 kg/ha application of nitrogen fertilizer, the level of NO_3_^−^−N increased with the increased application rate of biochar and was observed to be significantly higher under treatment B1200N126 (29.82 and 18.37 mg/kg) compared to B0N126, in both the pot and field experiments, respectively (LSD, *p* < 0.05; **Table 1**). These results suggested that the contents of NO_3_^−^−N decreased under the high application rate of biochar and low nitrogen level but increased with the increased application rate of biochar and nitrogen fertilizer.

**Table 1:** Effects of various combinations of biochar and nitrogen fertilizer on contents of soil organic carbon (SOC), total nitrogen (TN), carbon/nitrogen ratio (C/N), soil nitrate nitrogen (NO_3_^−^−N), and ammonium nitrogen (NH_4_^+^−N) of tobacco soil.

Experimental site	Treatments	SOC(g/kg)	TN(g/kg)	C/N	NO_3_^–^-N(mg/kg)	NH_4_^+^-N(mg/kg)
Pot experiment	B0N105	18.37 ± 0.09d	2.04 ± 0.04d	9.02 ± 0.15e	19.78 ± 1.00c	12.67 ± 1.72a
	B600N105	22.63 ± 0.55c	2.25 ± 0.04c	10.08 ± 0.34c	29.82 ± 1.45a	13.59 ± 0.9a
	B1200N105	30.04 ± 0.26a	2.24 ± 0.02c	13.39 ± 0.05a	22.46 ± 1.19b	14.05 ± 0.82a
	B0N126	17.89 ± 0.59d	2.06 ± 0.03d	8.69 ± 0.39e	20.66 ± 1.38bc	13.23 ± 0.67a
	B600N126	22.46 ± 0.45c	2.35 ± 0.01b	9.57 ± 0.15d	31.46 ± 0.97a	13.29 ± 0.74a
	B1200N126	29.11 ± 0.31b	2.43 ± 0.03a	12 ± 0.17b	29.82 ± 1.03a	13.22 ± 0.81a
Field experiment	B0N105	14.29 ± 0.23d	1.24 ± 0.03c	11.52 ± 0.24e	12.66 ± 0.34c	2.79 ± 0.05a
	B600N105	18.8 ± 0.68c	1.35 ± 0.02b	13.93 ± 0.71c	18.27 ± 0.34a	2.75 ± 0.03a
	B1200N105	24.07 ± 0.63a	1.38 ± 0.04b	17.44 ± 0.23a	11.37 ± 0.54d	2.73 ± 0.05a
	B0N126	13.92 ± 0.15d	1.3 ± 0.03d	10.71 ± 0.11f	13.22 ± 0.6c	2.77 ± 0.03a
	B600N126	20.75 ± 0.5b	1.57 ± 0.02a	13.25 ± 0.45d	16.87 ± 0.53b	2.75 ± 0.04a
	B1200N126	23.57 ± 0.34a	1.58 ± 0.04a	14.88 ± 0.13b	18.37 ± 0.26a	2.74 ± 0.03a

B0, B600, and B1200 represent 0, 600, and 1800 kg/ha biochar application rates, respectively. N105 and N126 represent 105 and 126 kg/ha application rates of pure nitrogen, respectively. The significant difference among treatments after specific days of post-transplantation is shown by different lowercase letters within a column according to the least significant difference test (LSD; p < 0.05).

### 3.2 Effect of different C/N ratios on SMC, SMN, and SMC/SMN ratios

The application of biochar (0, 600, and 1200 kg/ha) under two different levels (105 and 126 kg/ha) of nitrogen fertilizer had a significant impact on the contents of SMC, SMN, and SMC/SMN ratio (LSD, *p* < 0.05; **Table 2**). In both experiments (pot and field), the SMC and SMN first increased and then decreased with the increased application rate of biochar under 105 kg/ha application of nitrogen fertilizer but increased with the increased application rate of biochar under 126 kg/ha application of nitrogen fertilizer. The SMC (140.87 and 128.15 mg/kg) and SMN (18.67 and 14.57 mg/kg) were found significantly higher under treatment B1200N126 compared with B0N105 and B0N126, in both the pot and field experiments, respectively (LSD, *p* < 0.05; **Table 2**). The SMC under treatment B1200N126 increased by 61.92% and 70.38% in the pot experiment and increased by 78.93% and 75.40% in the field experiment compared with B0N105 and B0N126, respectively. The SMC/SMN ratio of flue-cured tobacco plants was found to be significantly higher under treatment B1200N105 (8.38 and 9.35) compared with B0N105 and B0N126, in both the pot and field experiments, respectively (LSD, *p* < 0.05; **Table 2**). The SMC/SMN ratio of flue-cured tobacco plants decreased under treatment B1200N126 with the increased biochar and nitrogen fertilizer application rate compared with B1200N105 (LSD, *p* > 0.05; **Table 2**).

**Table 2:** Effects of different biochar and nitrogen fertilizer ratios on contents of soil microbial biomass of carbon (SMC), soil microbial biomass of nitrogen (SMN), and SMC/SMN ratio of tobacco soil.

Experimental site	Treatments	SMC (mg/kg)	SMN (mg/kg)	SMC/SMN
Pot experiment	B0N105	87.00 ± 9.95c	13.63 ± 0.47b	6.40 ± 0.87c
	B600N105	126.46 ± 3.15b	17.70 ± 0.60d	7.15 ± 0.39b
	B1200N105	117.26 ± 1.08b	14.00 ± 0.33c	8.38 ± 0.27a
	B0N126	82.68 ± 3.83c	15.04 ± 0.49c	5.51 ± 0.44c
	B600N126	138.74 ± 6.09a	18.91 ± 0.83a	7.35 ± 0.56b
	B1200N126	140.87 ± 9.48a	18.67 ± 0.21a	7.54 ± 0.45ab
Field experiment	B0N105	71.62 ± 6.42d	9.72 ± 0.86c	7.37 ± 0.23c
	B600N105	109.61 ± 2.48b	13.15 ± 0.34b	8.34 ± 0.27b
	B1200N105	92.14 ± 3.68c	9.86 ± 0.32c	9.35 ± 0.26a
	B0N126	73.06 ± 4.07d	10.23 ± 0.55c	7.15 ± 0.49c
	B600N126	126.18 ± 4.14a	14.46 ± 0.28a	8.73 ± 0.13b
	B1200N126	128.15 ± 3.95a	14.57 ± 0.47a	8.79 ± 0.09b

B0, B600, and B1200 represent 0, 600, and 1800 kg/ha biochar application rates, respectively. N105 and N126 represent 105 and 126 kg/ha application rates of pure nitrogen, respectively. The significant difference among treatments after specific days of post-transplantation is shown by different lowercase letters within a column according to the least significant difference test (LSD; p < 0.05).

### 3.3 Influence of different C/N ratios on soil enzymatic activities

Soil enzymatic activities, including S-UE, S-SC, S-CAT, and S-ACP, significantly changed under the different C/N ratios in both the pot and field experiments (LSD, *p* < 0.05; **Table 3**). In both the pot and field experiments, the activities of S-UE, S-SC, S-CAT, and S-ACP first increased and then decreased with the increased biochar application rate under 105 kg/ha of nitrogen fertilizer. However, soil enzymatic activities increased with the increased biochar application rate under 126 kg/ha application of nitrogen fertilizer (**Table 3**). The activities of S-UE, S-SC, and S-CAT were found to be significantly higher under treatment B1200N126 than B0N105 and B0N126 in both experiments (LSD, *p* < 0.05; **Table 3**), except for S-CAT in the field experiment in which no significant difference was observed among the treatments (LSD, *p* > 0.05; **Table 3**). The activity of S-UE, S-SC, and S-CAT under treatment B1200N126 were increased by 53.33%, 64.82%, and 20.79%, respectively, compared with B0N105 and increased by 43.75%, 58.83%, and 22.54%, respectively, compared with B0N126 in the pot experiment. In the field experiment, the activity of S-UE and S-SC under treatment B1200N126 were increased by 86.44% and 53.37%, respectively, relative to B0N105 and increased by 77.41% and 41.51%, respectively, compared to B0N126. The activity of S-ACP was found to be significantly higher under treatment B1200N126 compared with B0N105 and B0N126 in pot and field experiments (LSD, *p* < 0.05; **Table 3**). The activity of S-ACP under treatment B1200N126 was increased by 42.05%, 132.25%, and 36.94%, 60% as compared with B0N105 and B0N126 in pot and field experiments, respectively.

**Table 3:** Impact of various combinations of biochar and nitrogen fertilizer on soil enzymatic activities.

Experimental site	Treatments	S-UE [mg/(d·g)]	S-ACP [umol/(d·g)]	S-CAT [umol/(min·g)]	S-SC [mg/(d·g)]
Pot experiment	B0N105	0.90 ± 0.03c	1.07 ± 0.04c	14.00 ± 0.68c	7.42 ± 0.47d
	B600N105	1.16 ± 0.04b	1.50 ± 0.04a	15.59 ± 0.72b	10.33 ± 0.47c
	B1200N105	0.88 ± 0.04c	1.28 ± 0.05b	12.02 ± 0.75d	5.85 ± 0.46e
	B0N126	0.96 ± 0.05c	1.11 ± 0.02c	13.80 ± 0.21c	7.70 ± 0.55d
	B600N126	1.23 ± 0.06b	1.54 ± 0.05a	16.78 ± 0.43a	11.10 ± 0.13b
	B1200N126	1.38 ± 0.08a	1.52 ± 0.06a	16.91 ± 0.51a	12.23 ± 0.34a
Field experiment	B0N105	0.59 ± 0.03d	0.31 ± 0.06c	11.56 ± 0.31a	4.89 ± 0.38b
	B600N105	0.82 ± 0.04c	0.67 ± 0.01a	11.90 ± 0.73a	7.64 ± 0.57a
	B1200N105	0.64 ± 0.05d	0.44 ± 0.05b	11.66 ± 0.32a	3.44 ± 0.20c
	B0N126	0.62 ± 0.04d	0.45 ± 0.03b	11.89 ± 0.48a	5.30 ± 0.81b
	B600N126	0.94 ± 0.02b	0.70 ± 0.07a	12.15 ± 0.36a	7.32 ± 0.31a
	B1200N126	1.10 ± 0.03a	0.72 ± 0.09a	11.96 ± 0.79a	7.50 ± 0.48a

Urease (UE), Acid phosphatase (ACP), Catalase (CAT), and Sucrase (SC). B0, B600, and B1200 represent 0, 600, and 1800 kg/ha biochar application rates, respectively. N105 and N126 represent 105 and 126 kg/ha application rates of pure nitrogen, respectively. The significant difference among treatments after specific days of post-transplantation is shown by different lowercase letters within a column according to the least significant difference test (LSD; p < 0.05).

### 3.4 Dry matter accumulation under different application rates of C/N ratio

The effect of different C/N ratios on the accumulation of dry matter in the whole and various parts (root, stem, and leaf) of tobacco plants was assessed in both experiments (**Table 4**). In both the pot and field experiments, the dry matter accumulation (g/plant) in whole and different parts (root, stem, and leaf) of flue-cured tobacco plants under 105 kg/ha of nitrogen fertilizer first increased and then decreased with the increased biochar application rate. While under 126 kg/ha application of nitrogen fertilizer, dry matter accumulation (g/plant) of flue-cured tobacco plants increased with the increased biochar application rate (**Table 4**). The accumulation of dry matter in the whole plant and different parts (root, stem, and leaf) of flue-cured tobacco plants was found to be significantly higher under treatment B1200N126 compared with B0N105 and B0N126 in the pot and field experiments (LSD, *p* < 0.05; **Table 4**). The whole plant dry matter accumulation under treatment B1200N126 was increased by 23.94% and 24.53% in the pot experiment and 31.57% and 23.97% in the field experiment, compared with B0N105 and B0N126. The accumulation of dry matter in different parts, such as the root, stem, and leaf, under treatment B1200N126 was increased by 31.94%, 20.16% and 23.69%, respectively, compared with B0N105, and 31.20%, 21.31%, and 24.32%, respectively, compared with B0N126 in the pot experiment. However, in the field experiment, the accumulation of dry matter in different parts (root, stem, and leaf) under treatment B1200N126 increased by 47.91%, 44.93%, and 23.17%, respectively, compared to B0N105, and 37.11%, 31.70%, and 18.26%, respectively, compared with B0N126. This suggests that the accumulation of dry matter in whole and different parts (root, stem, and leaf) of flue-cured tobacco plants increases under high application of nitrogen fertilizer (126 kg/ha) and biochar (1200 kg/ha), while it decreased under the excessive application of biochar (≧1200 kg/ha) and low nitrogen levels (105 kg/ha).

**Table 4:** Effects of different application ratios biochar and nitrogen fertilizer on dry matter accumulation and distribution of tobacco plants.

Experimental site	Treatments	Dry matter accumulation(g/plant)	Dry matter accumulation (g/plant)			Dry matter distribution ratio (%)		
			Root	Stem	Leaf	Root	Stem	Leaf
Pot experiment	B0N105	104.26 ± 1.67c	17.91 ± 0.74c	34.73 ± 0.37c	51.62 ± 1.05c	17.18 ± 0.43a	33.32 ± 0.68a	49.50 ± 0.40a
	B600N105	117.22 ± 1.00b	21.31 ± 1.56b	38.32 ± 0.61b	57.59 ± 1.21b	18.18 ± 1.31a	32.69 ± 0.66a	49.13 ± 0.83a
	B1200N105	101.04 ± 2.14c	18.92 ± 0.84c	34.54 ± 0.87c	47.58 ± 1.06c	18.73 ± 0.71a	34.18 ± 0.58a	47.09 ± 0.15b
	B0N126	103.77 ± 2.03c	18.01 ± 0.07c	34.40 ± 1.20c	51.36 ± 1.23c	17.36 ± 0.39a	33.14 ± 0.68a	49.49 ± 0.63a
	B600N126	129.02 ± 6.92a	22.65 ± 1.48ab	42.55 ± 1.40a	63.82 ± 3.41a	17.58 ± 1.20a	33.01 ± 1.26a	49.41 ± 1.59a
	B1200N126	129.22 ± 5.91a	23.63 ± 0.99a	41.73 ± 1.07a	63.85 ± 3.49a	18.27 ± 0.76a	32.33 ± 1.34a	49.40 ± 0.59a
Field experiment	B0N105	451.25 ± 23.65d	44.91 ± 3.77d	123.02 ± 12.15b	283.32 ± 11.30cd	9.94 ± 0.41c	27.23 ± 1.47bc	62.83 ± 1.88a
	B600N105	511.66 ± 17.17b	59.06 ± 6.48b	130.43 ± 10.16b	322.17 ± 2.13b	11.53 ± 0.95ab	25.47 ± 1.23c	63.01 ± 1.71a
	B1200N105	458.47 ± 2.78cd	55.99 ± 1.76c	131.64 ± 6.21b	270.84 ± 6.47d	12.21 ± 0.33a	29.22 ± 1.43ab	60.12 ± 1.35b
	B0N126	478.89 ± 8.04c	48.45 ± 2.34b	135.38 ± 1.04b	295.06 ± 6.85c	10.11 ± 0.37c	28.28 ± 0.58ab	61.61 ± 0.57a
	B600N126	590.80 ± 6.32a	66.40 ± 3.00a	180.80 ± 5.11a	343.61 ± 8.06a	11.24 ± 0.40b	30.61 ± 1.06a	58.16 ± 1.00b
	B1200N126	593.69 ± 9.14a	66.43 ± 0.56a	178.29 ± 4.65a	348.96 ± 9.05a	11.19 ± 0.26b	30.03 ± 0.76a	58.77 ± 0.82b

B0, B600, and B1200 represent 0, 600, and 1800 kg/ha biochar application rates, respectively. N105 and N126 represent 105 and 126 kg/ha application rates of pure nitrogen, respectively. The significant difference among treatments after specific days of post-transplantation is shown by different lowercase letters within a column according to the least significant difference test (LSD; p < 0.05).

### 3.5 Effect of different C/N ratios on bacterial community assembly of tobacco plants

A total of 18 rhizosphere samples from six different treatments (three biological replicates per treatment) were processed on an Illumina MiSeq platform for high-throughput amplicons sequencing of the 16S *rRNA* V3-V4 variable region of bacteria. The amplicons sequencing resulted in a total of 2,235,225 raw reads, with an average of 124,179 reads per sample (**Table S3**). After Chimera’s removal and quality control, 1,936,376 effective reads (107,576 reads per sample) were obtained with an average length of 461 bp/sample (**Table S3**). These effective reads were clustered into 55,469 OTUs, averaging 3,082 OTUs per sample (**Table S3**).

### 3.6 Bacterial community diversity, structure, and OTUs distribution under different application rates of C/N ratio

**Figure 1:**
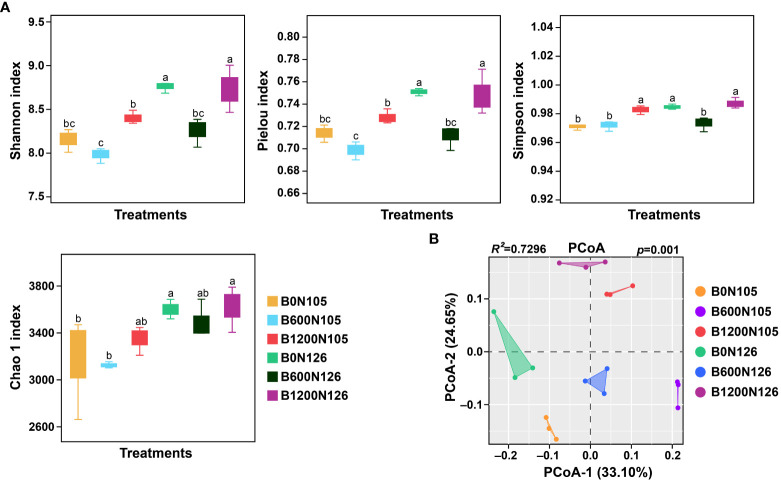
Impact of different application rates of carbon/nitrogen on diversity and structure of tobacco plant rhizosphere bacterial communities. **(A)** alpha diversity indices of rhizosphere bacterial communities under different treatments. **(B)** Principal coordinate analysis based on Bray–Curti’s dissimilarity matrix shows the changes in bacterial community structure under different treatments. The lowercase letters on the error bars represent the significant difference among treatments according to the least significant difference test at *p* < 0.05. B0, B600, and B1200 represent 0, 600, and 1800 kg/ha biochar application rates, respectively. N105 and N126 represent 105 and 126 kg/ha application rates of pure nitrogen, respectively.

Firstly the alpha diversity indices and beta diversity of bacterial communities were calculated under different application rates of C/N ratio (**Figure 1**). The alpha diversity indices (Shannon, Simpson, Pielou, and Chao1) of bacterial communities were significantly changed under different application rates of C/N ratio (**Figure 1** and **Table S4**). Shannon and Pielou indexes had substantially higher values under treatments B0N126 and B1200N126 compared with B0N125, B600N126, B1200N125, and B600N126 (LSD, *p* < 0.05; **Figure 1A**). Compared to B0N125, B600N125, and B600N126, treatments B1200N125, B0N126, and B1200N126 had significantly higher Simpson index values **(**LSD, *p* < 0.05), while no significant difference was observed among B1200N125, B0N126, and B1200N126 (LSD, *p* > 0.05; **Figure 1A**). The values of the Chao 1 index were increased significantly under treatments B0N126 and B1200N126 compared to B0N125 and B600N125 (LSD, *p* < 0.05), whereas no significant difference was found compared to B1200N125 and B600N126 (LSD, *p* > 0.05; **Figure 1A**). Bray–Curti’s dissimilarity matrix was used to calculate the variations in the structure of bacterial communities (beta diversity) under treatments, and results were visualized by PCoA. PCoA results showed a clear separation among the treatments, where PCoA-1 and PCoA-2 showed a total of 33.01% and 24.65% variations in the structure of bacterial community (**Figure 1B**). Furthermore, PERMANOVA analysis based on pairwise interactions between bacterial communities demonstrated that the overall structure of rhizosphere bacterial communities significantly changed under different treatments (*R^2 =^* 0.7296, *p* = 0.001, **Table S5**).

**Figure 2:**
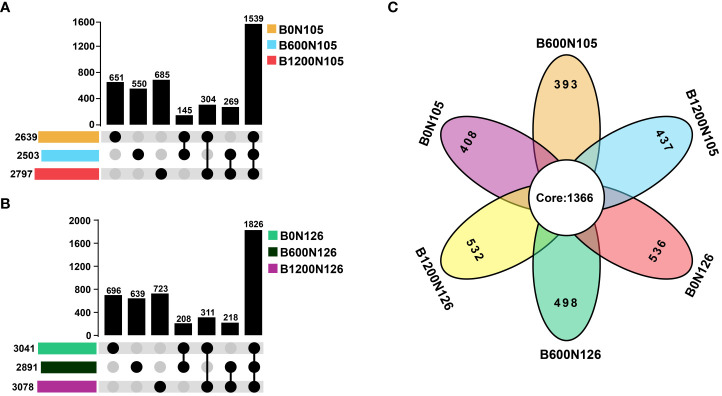
UpSet plots and Venn diagrams show the distribution of operational taxonomic units (OTUs) under different application rates of carbon/nitrogen ratios. **(A, B)** UpSet plots display the interaction between shared and unique OTUs under different treatments. **(C)** The Venn diagram illustrates the core and unique OTUs among the treatments. B0, B600, and B1200 represent 0, 600, and 1800 kg/ha biochar application rates, respectively. N105 and N126 represent 105 and 126 kg/ha application rates of pure nitrogen, respectively.

An OTUs distribution analysis confirmed the shared and unique OTUs among the treatments (**Figure 2**). UpSet plots displayed the interaction between shared and unique OTUs at different application rates of biochar (0, 600, and 1200 kg/ha) under two levels of nitrogen fertilizer 105 kg/ha (**Figure 2A**) and 126 kg/ha (**Figure 2B**). According to the results of UpSet plots, a significant difference was observed for shared and unique OTUs among the treatments under different application rates of biochar (0, 600, and 1200 kg/ha) at the same level of nitrogen fertilizer (105 and 126 kg/ha). The maximum number of unique OTUs was recorded under treatments B1200N105 (685 OTUs) and B1200N126 (723 OTUs) (**Figures 2A, B**). A Venn diagram further confirmed that the difference in the alpha and beta diversity indices of bacterial communities among the treatments might be due to common and unique OTUs (**Figure 2C**). These results suggest that alpha-beta diversity indices and OTUs distribution of bacterial communities significantly changed under different application rates of C/N ratio and were found to be significantly higher in treatment B1200N126 under high application of biochar (1200 kg/ha) and nitrogen fertilizer (126 kg/ha).

### 3.7 Bacterial community composition under different application rates of C/N ratio

**Figure 3:**
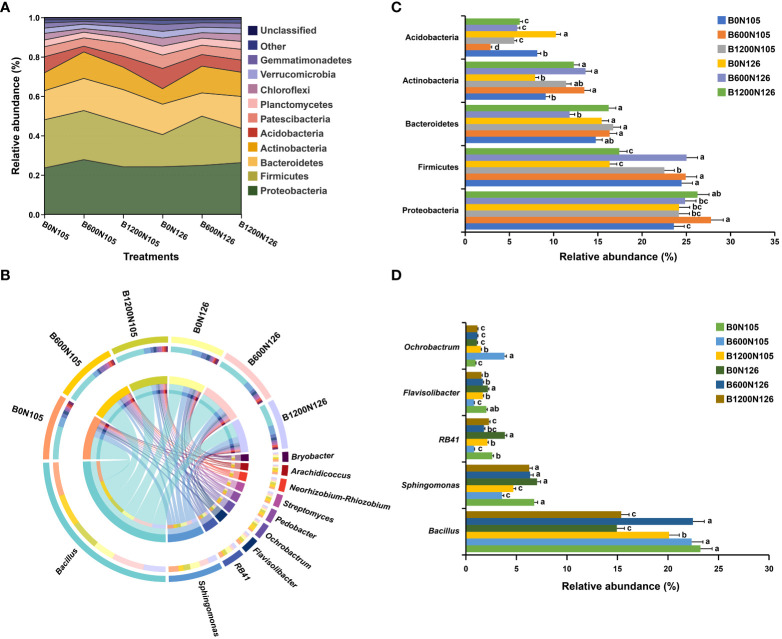
Relative abundance analysis of most abundant bacterial phylum and genus under different biochar and nitrogen fertilizer application ratios. River map **(A)** and chord diagram **(B)** showing the relative abundance of the top 10 bacterial phyla and genera under different treatments. Bar plots representing the significant difference among the most abundant bacterial phylum **(C)** and genus **(D)** among the treatments. According to the least significant difference test at *p* < 0.05, different small letters on the error bars show the difference among treatments. B0, B600, and B1200 represent 0, 600, and 1800 kg/ha biochar application rates, respectively. N105 and N126 represent 105 and 126 kg/ha application rates of pure nitrogen, respectively.

The bacterial community composition at the phylum and genus level significantly changed under different application rates of C/N ratio (**Figure 3** and **Tables S6**, **S7**). The relative abundance (RA) of the top 10 bacterial communities at the phylum and genus level is shown in the river map (**Figure 3A** and **Table S6**) and chord diagram (**Figure 3B** and **Table S7**). The phyla Actinobacteria, Acidobacteria, Bacteroidetes, Firmicutes, Proteobacteria, and Patescibacteria were present in high RA in all rhizosphere soil samples, dominated the rhizosphere soil bacterial communities, and accounted for around 85.40% of total soil bacteriome. Furthermore, RA bar plots were constructed to confirm the significant difference in the RA of specific phylum and genera among the treatments (**Figures 3C, D**). The RA of Actinobacteria, Bacteroidetes, and Proteobacteria were significantly increased, and the RA of Acidobacteria and Firmicutes significantly decreased in the rhizosphere soil of flue-cured tobacco plants under treatment B1200N125 (LSD, *p* < 0.05; **Figure 3C**). Furthermore, RA analysis at the genus level revealed that the RA of *Bacillus* was significantly increased under treatments B0N105, B600N105, and B600N126 (LSD, *p* < 0.05), and the RA of *Sphingomonas* was decreased considerably under B600N105 and B1200N105 (LSD, *p* < 0.05; **Figure 3D**). The RA of *RB41* and *Flavisolibacter* was significantly increased under B0N126, while the RA of *Ochrobactrum* was increased dramatically under B600N106 (LSD, *p* < 0.05; **Figure 3D**).

### 3.8 Characteristics of bacterial co-occurrence network and redundancy analysis

**Figure 4:**
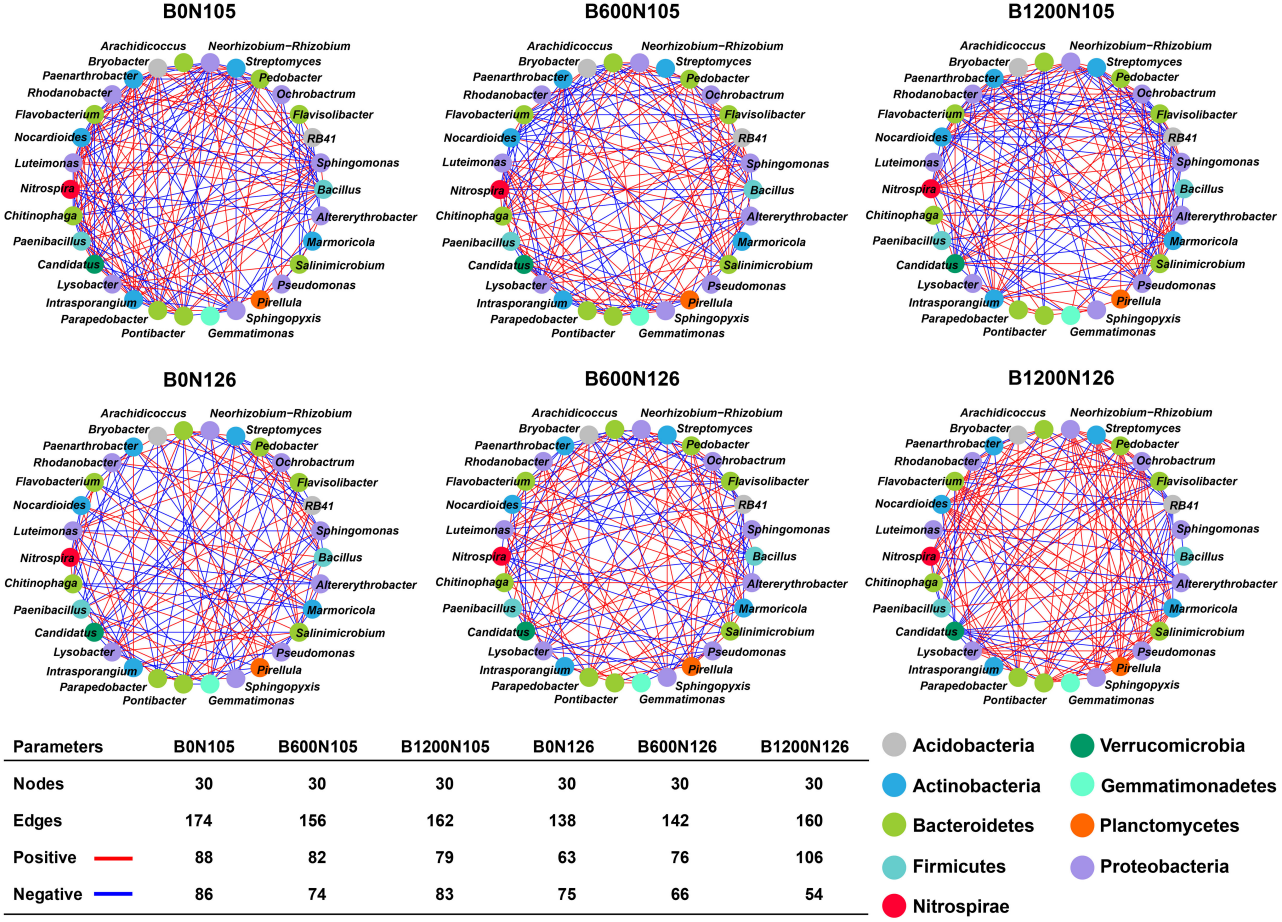
Co-occurrence network analysis of rhizosphere bacterial communities at phylum level under different treatments. Nodes represent the bacterial phylum, and edges show the positive (red line) and negative (red lines) correlations among the bacterial phyla within a treatment. B0, B600, and B1200 represent 0, 600, and 1800 kg/ha biochar application rates, respectively. N105 and N126 represent 105 and 126 kg/ha application rates of pure nitrogen, respectively.

Bacterial co-occurrence network analysis was performed according to “spearmen correlation (*p* < 0.05 and correlation coefficient > 0.9) for most abundant bacterial genera within phylum under different treatments (**Figure 4**). Nodes represent the specific bacterial phylum and edges display a pairwise correlation (positive; red lines and negative; blue lines) between the nodes. Together, nodes and edges show a biochemical interaction among the bacterial communities within the network. The number of nodes (30) was found to be the same under different treatments, and no significant difference was observed among the treatments. The number of edges decreased in the order B0N105 (174) > B1200N105 (162) > B1200N126 (160) > B600N105 (156) > B600N126 (142) > B0N126 (138). Analysis of pairwise correlation among the bacterial communities within a treatment showed the highest positive (106) and fewest negative (54) correlations among the bacterial communities under treatment B1200N126 compared with other treatments (**Figure 4**). No significant difference was observed for positive 88, 82, 79, 63, and 76 edges and negative 86, 74, 83, 75, and 66 edges among the nodes under treatments B0N105, B600N105, B1200N105, B0N126, and B600N126 (**Figure 4**).

**Figure 5:**
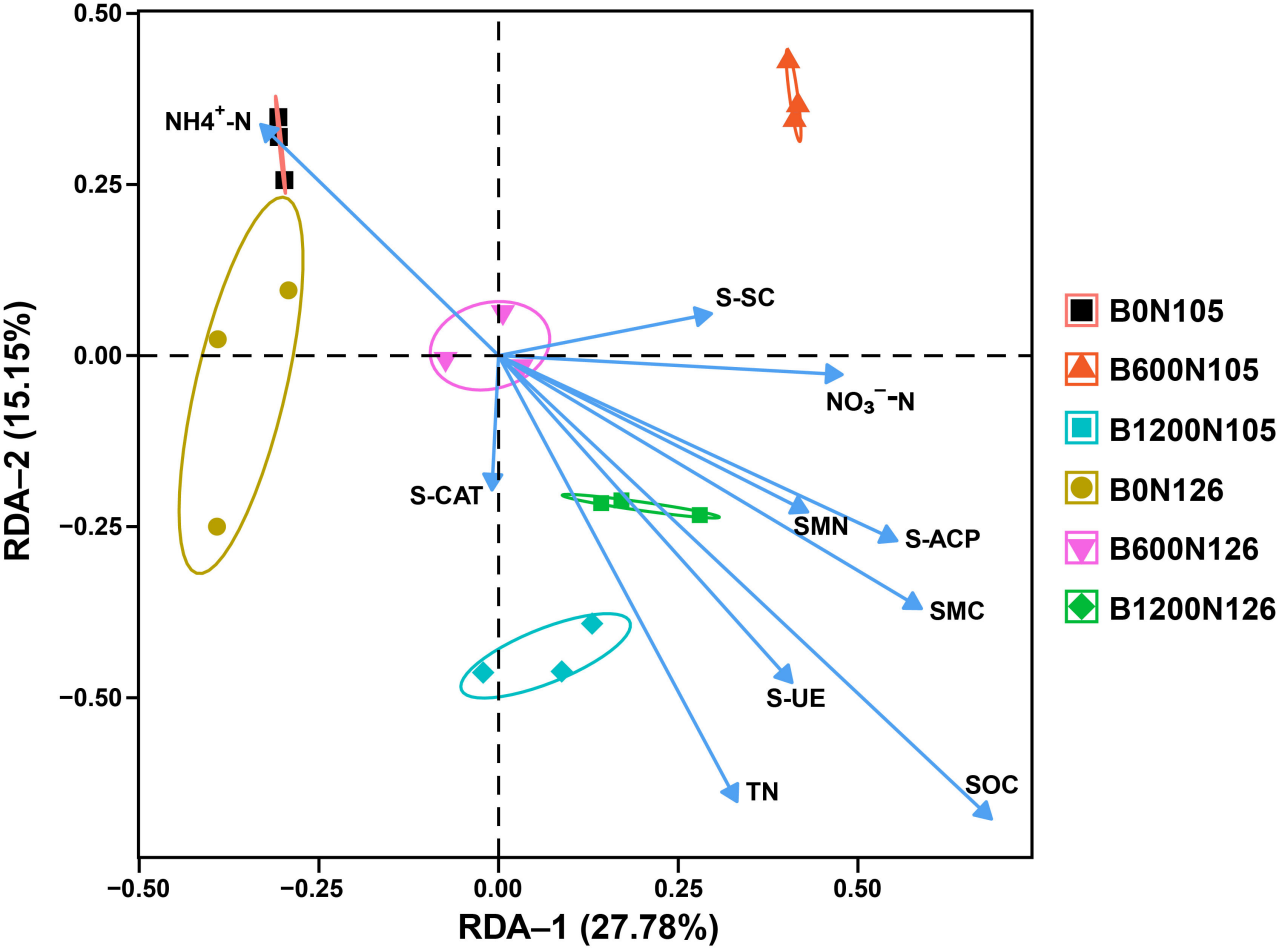
Redundancy analysis shows the correlation between rhizosphere bacterial communities, soil physicochemical properties, soil enzymatic activities, and soil microbial biomass of carbon and nitrogen under different treatments. B0, B600, and B1200 represent 0, 600, and 1800 kg/ha biochar application rates, respectively. N105 and N126 represent 105, and 126 kg/ha application rates of pure nitrogen, respectively.

An RDA further confirmed that rhizosphere soil bacterial communities, soil physicochemical properties, enzymatic activity, SMC, and SMN were affected by different application rates of the C/N ratio. RDA displayed a total of 42.93% variation in rhizosphere soil bacterial communities, soil physicochemical properties, enzymatic activity, SMC, and SMN under different treatments (**Figure 5**). RDA results revealed that TN, SOC, S-UE, S-SC, S-CAT, S-ACP, NO_3_^−^-N, SMN, and SMN had a strong positive correlation with treatment B1200N126, while NH_4_^+^-N had a negative correlation with B1200N126 (**Figure 5**).

## 4 Discussion

This study aimed to evaluate the impact of the combined application of biochar and nitrogen fertilizer on soil properties, rhizosphere bacterial community, and biomass accumulation of flue-cured tobacco plants, which might be related to the biochar and nitrogen fertilizer dose and their interaction. The results showed that the appropriate application of biochar and nitrogen fertilizer significantly affected the accumulation of tobacco biomass. A proper biochar dose (600 kg/ha and 1200 kg/ha) under high nitrogen (N) fertilizer (126 kg/ha) was beneficial to the dry matter accumulation of tobacco plants and soil biochemical and microbial activities. However, a high dose of biochar (1200 kg/ha) under a low N fertilizer rate (105 kg/ha) was not conducive to the dry matter accumulation in tobacco plant and soil biochemical and microbial activities, which is in the agreement with previous studies (Ali et al., 2020; Sun et al., 2022). Biochar can improve soil’s physicochemical properties, nutrient availability, and utilization rate and promotes tobacco plants’ growth and dry matter accumulation (Ding et al., 2016; Zheng et al., 2021). This study shows that combining biochar and nitrogen fertilizer significantly increases the contents of soil organic carbon, soil total nitrogen, and soil carbon-nitrogen ratio. Our findings are consistent with the previous studies reporting that using biochar and mineral fertilizers simulate crop growth (Schulz and Glaser, 2012). We found that the soil NO_3_^−^−N concentration was higher under the combined application of biochar and nitrogen than that of single nitrogen fertilizer treatment. This indicates that the combined application of biochar and nitrogen fertilizer had a synergistic effect on soil nitrification, and it might be related to the adsorption of nitrification inhibitors (phenol and terpene) by biochar (Ball et al., 2010). In addition, biochar can significantly increase soil organic carbon, resulting in a higher carbon-nitrogen ratio, thereby enhancing soil nitrification and improving nitrogen bioavailability, increasing plants’ overall biomass accumulation (Zhao et al., 2020).

It was found that the high biochar dosage (1200 kg/ha) and low nitrogen levels (105 kg/ha) were not conducive to the dry matter accumulation in tobacco leaves and whole plants. Some reports have shown adverse or insignificant effects of biochar on crop growth and yield. For example, Li et al. (2022) found that when the concentration of tobacco stem biochar reached more than 4%, the soil nitrate-nitrogen content began to decline rapidly, and the dry matter accumulation of tobacco plants also reduced significantly than that of the control. Excessive biochar can inhibit crop growth due to nitrogen fixation caused by the high content of volatile components and toxic or harmful substances, reducing nutrient absorption and crop growth (Zhang et al., 2018; Song et al., 2020). The heavy metals in biochar are all below the threshold level of heavy metals which are considered damaging for plant growth, as reported by the International Biochar Initiative (IBI) and the European Biochar Certificate (EBC) (IBI, 2016). Therefore, nitrogen fixation may be a factor in limiting tobacco growth under higher biochar doses. Zheng et al. (2020) reported that high amounts of biochar may inhibit the growth of tobacco because at high C/N ratios, microbial biomass fixes nitrogen, thereby reducing nitrogen availability, which is in concurrence with our study. However, we found that high doses of biochar (1200 kg/ha) and nitrogen (126 kg/ha) treatments increased the biomass of tobacco plants, which was related to increasing the amount of nitrogen fertilizer and reducing the C/N ratio (Xia et al., 2022).

Soil enzyme activity can sensitively and accurately reflect the changes in soil quality and is closely related to soil physicochemical properties, nutrient contents, and fertilization methods (Datta et al., 2017; Zhou et al., 2020). We found that the rational combination of biochar and nitrogen fertilizer significantly increased the enzyme activities of soil urease, soil invertase, soil acid phosphatase, and hydrogen peroxide, which is in accordance with the results of previous studies (Wang et al., 2015; Elzobair et al., 2016). Similarly, biochar stimulates soil enzyme activity and affects bacterial community abundance by increasing soil organic carbon and available nitrogen, which is consistent with previous reports (Li et al., 2016). Applying biochar (600 kg/ha) and nitrogen (105 kg/ha and 126 kg/ha) significantly increased soil organic carbon and available nitrogen contents, which may be partly due to the increase in urease activity. Previous studies reported that biochar application may inhibit enzymatic reactions by occupying binding sites (Liu et al., 2018; Ali et al., 2021), which is also related to biochar and soil types. However, further in-depth analysis is needed for different situations. A suitable nitrogen fertilizer application rate should be maintained when biochar and nitrogen fertilizer are applied together. This experiment showed that the soil enzyme activity was decreased when the amount of biochar was too high (1200 kg/ha), and the amount of nitrogen fertilizer was too low (105 kg/ha). This might be due to excessive biochar and low nitrogen fertilizer increased the C/N ratio, inhibiting the soil microbial activity and reducing the number of rhizosphere microorganisms (Zheng et al., 2020). However, under high dosages of biochar (1200 kg/ha) and nitrogen fertilizer (126 kg/ha), the soil C/N ratio decreased, which enhanced the soil enzyme activity. These findings suggest that the key reason for the reduced enzyme activity might be related to the soil’s high C/N ratio.

Biochar can significantly increase the contents of microbial biomass carbon in the soil, mainly when applied with NPK fertilizer (Khan et al., 2021b). This study found that the appropriate amount of biochar (600 kg/ha) combined with nitrogen fertilizer significantly increased the contents of microbial biomass carbon and nitrogen. One possible explanation is that biochar has a porous structure and a large specific surface area, which can maintain and balance soil moisture, air, and nutrients, thereby improving the living conditions for microorganisms (Li et al., 2020). In addition, biochar may become a new carbon source for microorganisms after degradation in soil and promote microbial growth (Ullah et al., 2020). More importantly, the combination of biochar (600 kg/ha) and nitrogen fertilizer (105 kg/ha and 126 kg/ha) significantly increased the contents of soil microbial biomass carbon and nitrogen compared with the control (biochar 0 kg/ha and N fertilizer 105 kg/ha and 126 kg/ha). This can be attributed to the synergistic effect of biochar and fertilizer and may also be because biochar can adsorb nitrogen and other nutrients, creating a nutrient-rich microenvironment and providing ideal conditions for microbial growth (Ullah et al., 2020; Zhao et al., 2022). However, in this study, the content of soil microbial biomass carbon and nitrogen under the combined application of high biochar (1200 kg/ha) and low nitrogen fertilizer (105 kg/ha) was significantly lower than that in the combined application of high biochar (1200 kg/ha) and high nitrogen fertilizer (126 kg/ha), which may be related to the soil C/N ratio and needs further in-depth study.

Soil microorganisms are an integral part of the soil’s ecosystem (Dai et al., 2023). The abundance and composition of soil microbes affect the soil structure and nutrient cycle, thus affecting plant growth, development, yield, and quality (Zheng et al., 2021). This study found that applying biochar and nitrogen fertilizer increased the diversity and abundance of rhizosphere bacteriomes to a certain extent. This might be because the porous structure of biochar and its ability to adsorb water and fertilizer make it a suitable habitat for soil microorganisms, which enhances the abundance of the rhizosphere microbiome, and increased application of nitrogen fertilizer promotes the reproduction of microorganisms in the soil (Ali et al., 2020). In this study, compared with high biochar (1200 kg/ha) and low nitrogen (105 kg/ha) treatments, the high biochar (1200 kg/ha) and high nitrogen (126 kg/ha) significantly increased the diversity of rhizosphere bacterial communities. This shows that the appropriate application of biochar and nitrogen fertilizer has a more positive effect on promoting the diversity of rhizosphere bacteriome, which is mainly favorable to the species richness of rhizosphere microorganisms.

The changes in the structure and composition of microbial communities depend on the source of the biochar and soil type (Adams et al., 2021). It has been reported that biochar can significantly affect the abundance of soil bacterial communities, but different soil types and microbial species have different responses to biochar (Adams et al., 2021). This study found that after applying biochar and nitrogen fertilizer, Acidobacteria, Bacteroidetes, Firmicutes, Proteobacteria, and Patescibacteria were the dominant phylum. The relative abundance of Actinobacteria, Bacteroidetes, and Proteobacteria in the rhizosphere soil of flue-cured tobacco was significantly increased by a high amount of biochar (1200 kg/ha) and high nitrogen (126 kg/ha), while that of Acidobacteria and Firmicutes decreased. These results are in accordance with previous reports that soil amendment with biochar enhanced the relative abundance of Proteobacteria, Bacteroidetes, and Actinobacteria but reduces the relative abundance of Acidobacteria, Chloroflexi, and Gemmatimonadetes (Xu et al., 2016). A co-occurrence network analysis showed a lower negative correlation among the rhizosphere bacterial communities under higher biochar and nitrogen fertilizer application rates than other treatments.

Further, RDA analysis proved that soil physicochemical and enzymatic properties were positively correlated with higher biochar and nitrogen fertilizer application rates. This may be affected by synergistic effects, such as the co-metabolism or synthesis of these bacteria or their similar reaction patterns to biological, chemical, or physical variables and common niches (Ullah et al., 2020). However, the effect of biochar and nitrogen fertilizer on the rhizosphere bacterial community is a complex process, and the internal mechanism of microorganisms needs to be elucidated through long-term experiments.

## 5 Conclusions

In summary, we conclude that the combined application of biochar and nitrogen significantly increase the soil carbon-nitrogen pool and change the soil carbon/nitrogen ratio, influencing the abundance and composition of rhizosphere bacteriome. The appropriate application of biochar combined with nitrogen could significantly increase the soil microbial biomass carbon and nitrogen, soil enzymatic activities, and biomass of flue-cured tobacco plants. The combined application of high biochar (1200 kg/ha) and low nitrogen (105 kg/ha) increased the soil C/N ratio, reducing the soil enzymatic activity, bacterial diversity, and biomass accumulation. In contrast, higher application of biochar (1200 kg/ha) and high nitrogen (126 kg/ha) reduced the soil C/N ratio and enhanced soil enzymatic activity, soil physicochemical properties, and relative abundance of rhizosphere bacteriome, which increased the biomass accumulation of flue-cured tobacco plants. Future studies will focus on elucidating the effect of high biochar and nitrogen fertilizer application on dry matter accumulation and the rhizosphere bacteriome of tobacco plants under a long-term mono-cropping system.

## Data availability statement

The raw data related to 16S rRNA sequencing is deposited in the sequence read archive (SRA) in NCBI public database with BioProject No. PRJNA975294.

## Author contributions

ZZ conceived and designed the experiments. YY, CY, WZ, XZ, HL, and DY performed the experiments. YY, WA, and XZ collected and analyzed the data. YY and WA wrote the manuscript. WA and ZZ revised the manuscript. All authors contributed to the final draft of the manuscript. All authors have read and agreed to the published version of the manuscript.
