# Supplementary material for: Elucidating the impact of biochar with different carbon/nitrogen ratios on soil biochemical properties and rhizosphere bacterial communities of flue-cured tobacco plants
Source: Front Plant Sci. 2023 Sep 15;14:1250669. doi: 10.3389/fpls.2023.1250669 (PMC10543665; doi:10.3389/fpls.2023.1250669)
Supplement: Supplementary file 1 [file Table_1.doc]

**Elucidating the impact of biochar with different carbon/nitrogen ratio on soil biochemical properties and rhizosphere bacterial communities of flue-cured tobacco plants**

Yingfen Yang^1^, Chenghu Ye^2^, Wei Zhang^1^, Xiaohong Zhu^1^, Haohao Li^3^, Dehai Yang^4^, Waqar Ahmed^1^*, and Zhengxiong Zhao^1^*

^1^Yunnan Agricultural University, Kunming, Yunnan, China

^2^Yunnan Revert Medical and Biotechnology Co., Ltd., Kunming, Yunnan, China

^3^Kunming Branch of Yunnan Tobacco Company, Kunming, Yunnan, China

^4^Hongta Tobacco Group Limited Company, Dali, Yunnan, China

***Correspondence authors**

**Name:** Waqar Ahmed

**Email:** ahmed.waqar1083@yahoo.com

**Name:** Zhengxiong Zhao

**Email:** zhaozx0801@163.com

| **Table S1; Basic physicochemical properties of soil at both experimental sites.** | | | | | | | | |
| --- | --- | --- | --- | --- | --- | --- | --- | --- |
| Experimental  condition | Experimental  site | Soil type | pH | SOC  (g/kg) | TN  (g/kg) | AN  (mg/kg) | AP  (mg/kg) | AK  (mg/kg) |
| Pot experiment | Kunming | paddy soil | 6.73 | 18.49 | 1.95 | 148.22 | 42.54 | 251.2 |
| Field experiment | Qujing | red soil | 5.92 | 12.81 | 1.14 | 92 | 22.85 | 180.7 |
| **Here,** Soil organic carbon **(SOC)**, total nitrogen **(TN)**, available nitrogen **(AN)**, available phosphorus **(AP)**, and available potassium **(AK)**. | | | | | | | | |

| **Table S2; Basic physicochemical properties of biochar used in the experiments** |
| --- |
| The basic physicochemical properties of tobacco stem biochar were as follow: pH (10.16), total carbon (57.83%), total nitrogen (2.05%), total phosphorus (1.24%), total potassium (3.65%), total cation exchange (12.07 cmol/kg), electrical conductivity (3.68 ms/cm), bulk density (0.23 g/cm^3^), and ash contents (28.9%). |

| **Table S3; Sequencing data processing for variable regions of bacteria (16S; V3-V4)** | | | | | | |
| --- | --- | --- | --- | --- | --- | --- |
| **Sample ID** | **Raw reads (#)** | **Clean reads (#)** | **Effective reads (#)** | **N50** | **Effective reads (%)** | **OTUs** |
| **Bacteria (16S; V3-V4)** | | | | | | |
| B0N105-1 | 133942 | 133850 | 115587 | 461 | 86.3 | 3172 |
| B0N105-2 | 131507 | 131422 | 113655 | 461 | 86.43 | 3099 |
| B0N105-3 | 62968 | 62928 | 54706 | 461 | 86.88 | 2247 |
| B600N105-1 | 124263 | 124184 | 107157 | 461 | 86.23 | 2792 |
| B600N105-2 | 137382 | 137290 | 118520 | 461 | 86.27 | 2839 |
| B600N105-3 | 125227 | 125131 | 108366 | 461 | 86.54 | 2744 |
| B1200N105-1 | 135895 | 135796 | 117873 | 461 | 86.74 | 2958 |
| B1200N105-2 | 136964 | 136861 | 119266 | 461 | 87.08 | 3032 |
| B1200N105-3 | 129857 | 129771 | 111657 | 461 | 85.98 | 3148 |
| B0N126-1 | 121419 | 121351 | 104211 | 461 | 85.83 | 3227 |
| B0N126-2 | 121808 | 121718 | 105679 | 461 | 86.76 | 3331 |
| B0N126-3 | 128044 | 127974 | 111741 | 459 | 87.27 | 3409 |
| B600N126-1 | 123554 | 123462 | 107707 | 461 | 87.17 | 3136 |
| B600N126-2 | 128900 | 128798 | 111418 | 461 | 86.44 | 3349 |
| B600N126-3 | 110577 | 110502 | 95953 | 461 | 86.77 | 3066 |
| B1200N126-1 | 133459 | 133368 | 115635 | 461 | 86.64 | 3497 |
| B1200N126-2 | 127512 | 127428 | 110371 | 461 | 86.56 | 3337 |
| B1200N126-3 | 121947 | 121872 | 106874 | 461 | 87.64 | 3086 |
| Total | 2235225 | 2233706 | 1936376 |  |  | 55469 |
| Average | 124179 | 124095 | 107576 | 461 |  | 3082 |
| **Note:** Raw reads stands for original reads. Clean reads are raw reads that filter low quality and short length sequences. Effective reads are the sequence of reads that are finally used for subsequent analysis after filtering chimeras. Effective reads (%) represents the percentage of the number of effective reads to the number of raw reads. **Here;** 0 kg/ha of biochar + 105 kg/ha of pure nitrogen (B0N105), 600 kg/ha of biochar + 105 kg/ha of pure nitrogen (B600N105), 1200 kg/ha of biochar + 105 kg/ha of pure nitrogen (B1200N105), 0 kg/ha of biochar + 126 kg/ha of pure nitrogen (B0N126), 600 kg/ha of biochar + 126 kg/ha of pure nitrogen (B600N126), and 1200 kg/ha of biochar + 126 kg/ha of pure nitrogen (B1200N126). | | | | | | |

| **Table S4; Sequencing data processing for variable regions of bacteria (16S; V3-V4)** | | | | |
| --- | --- | --- | --- | --- |
| **Sample ID** | **Chao 1 index** | **Simpson index** | **Shannon index** | **Pielou index** |
| **Bacteria (16S; V3-V4)** | | | | |
| B0N105 | 3277.69±463.47b | 0.97±0.002b | 8.11±0.15bc | 0.7138±0.0078bc |
| B600N105 | 3208.56±31.15b | 0.97±0.004b | 7.97±0.08c | 0.6985±0.0081c |
| B1200N105 | 3460.26±118.23ab | 0.98±0.003a | 8.41±0.07b | 0.7280±0.0066b |
| B0N126 | 3731.15±90.69a | 0.98±0.001a | 8.68±0.04a | 0.7508±0.0033a |
| B600N126 | 3616.73±180.12ab | 0.97±0.005b | 8.22±0.16bc | 0.7111±0.0112bc |
| B1200N126 | 3730.14±209.14a | 0.99±0.004a | 8.74±0.27a | 0.7484±0.0203a |
| **Here;** 0 kg/ha of biochar + 105 kg/ha of pure nitrogen (B0N105), 600 kg/ha of biochar + 105 kg/ha of pure nitrogen (B600N105), 1200 kg/ha of biochar + 105 kg/ha of pure nitrogen (B1200N105), 0 kg/ha of biochar + 126 kg/ha of pure nitrogen (B0N126), 600 kg/ha of biochar + 126 kg/ha of pure nitrogen (B600N126), and 1200 kg/ha of biochar + 126 kg/ha of pure nitrogen (B1200N126).Significant differences among treatments represents by different small letters within a column according to LSD-test at *p < 0.05*. | | | | |
|
|
|

| **Table S5; Results of permutational multivariate analysis of variance (PERMANOVA) for bacterial communities based on Bray-Curtis distance matrix.** | | | | | | | |
| --- | --- | --- | --- | --- | --- | --- | --- |
| **Pairs** | **df** | **SumsOfSqs** | **MeanSqs** | **F.value** | **R^2^** | **Pvalue** | **significant** |
| **Bacteria (16S; V3-V4)** | | | | | | | |
| B0N105-vs-MB600N105 | 1 | 0.1758 | 0.1758 | 10.6854 | 0.7276 | 0.1 | ns |
| MB0N105-vs-MB1200N105 | 1 | 0.1595 | 0.1595 | 8.9283 | 0.6906 | 0.1 | ns |
| MB600N105-vs-MB1200N105 | 1 | 0.1203 | 0.1203 | 8.1264 | 0.6701 | 0.1 | ns |
| MB0N126-vs-MB600N126 | 1 | 0.1159 | 0.1159 | 4.7839 | 0.5446 | 0.1 | ns |
| MB0N126-vs-MB1200N126 | 1 | 0.1191 | 0.1191 | 4.1213 | 0.5075 | 0.1 | ns |
| MB600N126-vs-MB1200N126 | 1 | 0.111 | 0.111 | 5.4951 | 0.5787 | 0.1 | ns |
| MB0N105-vs-MB0N126 | 1 | 0.083 | 0.083 | 3.1654 | 0.4418 | 0.1 | ns |
| MB600N105-vs-MB600N126 | 1 | 0.1061 | 0.1061 | 7.3403 | 0.6473 | 0.1 | ns |
| MB1200N105-vs-MB1200N126 | 1 | 0.0631 | 0.0631 | 3.0714 | 0.4343 | 0.1 | ns |
| MB600N105-vs-MB1200N126 | 1 | 0.1843 | 0.1843 | 9.6356 | 0.7067 | 0.1 | ns |
| MB0N105-vs-MB600N105-vs-MB1200N105 | 2 | 0.3037 | 0.1519 | 9.2751 | 0.7556 | 0.001 | ** |
| MB0N126-vs-MB600N126-vs-MB1200N126 | 2 | 0.2306 | 0.1153 | 4.7188 | 0.6113 | 0.007 | ** |
| MB0N105-vs-MB600N105-vs-MB1200N105-vs-MB0N126-vs-MB600N126-vs-MB1200N126 | 5 | 0.6608 | 0.1322 | 6.4767 | 0.7296 | 0.001 | ** |
| **Here;** 0 kg/ha of biochar + 105 kg/ha of pure nitrogen (B0N105), 600 kg/ha of biochar + 105 kg/ha of pure nitrogen (B600N105), 1200 kg/ha of biochar + 105 kg/ha of pure nitrogen (B1200N105), 0 kg/ha of biochar + 126 kg/ha of pure nitrogen (B0N126), 600 kg/ha of biochar + 126 kg/ha of pure nitrogen (B600N126), and 1200 kg/ha of biochar + 126 kg/ha of pure nitrogen (B1200N126). Asterisks indicates significant differences. at **p* < 0.05, ***p* < 0.01, and ^ns^*p* > 0.05. | | | | | | | |

| **Table S6; Relative abundance of top 11 bacterial phyla in rhizosphere soil samples under different treatments (±SEM, n=3/treatment)** | | | | | | |
| --- | --- | --- | --- | --- | --- | --- |
| **Phylum** | **B0N105** | **B600N105** | **B1200N105** | **B0N126** | **B600N126** | **B1200N126** |
| **Bacteria (16S; V3-V4)** | | | | | | |
| Proteobacteria | 23.5845±0.7998c | 27.787±0.7042a | 24.1479±1.3355bc | 24.1785±0.98bc | 24.8578±1.3175bc | 26.2639±1.6884ab |
| Firmicutes | 24.4682±1.1123a | 24.9298±1.0024a | 22.516±2.4439b | 16.3328±2.4635c | 25.0294±2.5849a | 17.4269±2.8974c |
| Bacteroidetes | 14.7592±2.5533ab | 16.3327±1.9774a | 16.7354±1.1088a | 15.4367±1.3393a | 11.8023±0.0802b | 16.2219±2.1203a |
| Actinobacteria | 9.0954±0.7994b | 13.4792±1.5105a | 11.4058±0.4786ab | 7.9072±1.7762b | 13.6034±0.6970a | 12.286±2.9193a |
| Acidobacteria | 8.1454±0.8965b | 2.8625±0.0783d | 5.5496±1.2212c | 10.2597±0.5817a | 5.8869±1.0461c | 6.1723±1.6120c |
| Patescibacteria | 5.0674±0.7560bc | 4.2665±0.7200c | 6.5757±0.5539a | 6.7967±0.6338a | 4.7445±0.5173bc | 5.4707±0.1793b |
| Planctomycetes | 3.4565±0.2138bc | 2.8717±0.8455c | 2.8618±0.0306c | 4.5298±0.4829a | 3.5861±0.3071bc | 4.0344±0.5695ab |
| Chloroflexi | 3.3463±0.2880b | 1.7998±0.1658d | 2.6823±0.2964d | 4.1095±0.2634a | 2.7476±0.2564c | 3.8012±0.1909a |
| Verrucomicrobia | 2.6919±0.2394bc | 2.2894±0.2390c | 2.7743±0.2636b | 3.42±0.1463a | 2.8077±0.0488b | 2.906±0.3821b |
| Gemmatimonadetes | 2.6414±0.2655b | 1.3651±0.0764d | 1.9624±0.1687c | 3.5236±0.3346a | 2.2333±0.1384bc | 2.567\±0.3708b |
| Other | 1.5007±0.0782d | 0.9613±0.0322e | 1.7349±0.0855bc | 2.0674±0.0786a | 1.5265±0.1382cd | 1.8211±0.2379b |
| **Here;** 0 kg/ha of biochar + 105 kg/ha of pure nitrogen (B0N105), 600 kg/ha of biochar + 105 kg/ha of pure nitrogen (B600N105), 1200 kg/ha of biochar + 105 kg/ha of pure nitrogen (B1200N105), 0 kg/ha of biochar + 126 kg/ha of pure nitrogen (B0N126), 600 kg/ha of biochar + 126 kg/ha of pure nitrogen (B600N126), and 1200 kg/ha of biochar + 126 kg/ha of pure nitrogen (B1200N126).Significant differences among treatments represents by different small letters within a column according to LSD-test at *p < 0.05*. | | | | | | |

| **Table S7; Relative abundance of 20 most dominant bacterial in rhizosphere soil samples under different treatments.** | | | | | | |
| --- | --- | --- | --- | --- | --- | --- |
| **Genus** | **B0N105** | **B600N105** | **B1200N105** | **B0N126** | **B600N126** | **B1200N126** |
| **Bacterial (16S; V3-V4)** | | | | | | |
| *Bacillus* | 23.2053±1.0763a | 22.3392±1.1118a | 20.1068±2.3693b | 14.939±2.6274c | 22.4669±2.2888a | 15.3732±3.1227c |
| *Sphingomonas* | 6.7576±0.5023a | 3.5797±0.2367c | 4.6963±0.2406c | 7.0413±0.9022a | 6.3467±0.1338a | 6.2631±0.4808a |
| *RB41* | 2.6053±0.6279b | 0.8112±0.0645c | 2.1198±0.5702b | 3.86±0.8314a | 1.7922±0.2998bc | 2.279±0.9457b |
| *Flavisolibacter* | 2.0166±0.3159ab | 0.8002±0.1274c | 1.6704±0.0299b | 2.1699±0.0666a | 1.6797±0.2086b | 1.5459±0.3263b |
| *Ochrobactrum* | 0.9527±0.0901c | 3.8139±0.2642a | 1.4706±0.2148c | 1.088±0.1477b | 1.1341±0.1899bc | 1.1456±0.2445bc |
| *Pedobacter* | 1.3152±0.0717c | 2.0013±0.132a | 1.7036±0.1494b | 1.0685±0.1009c | 1.2721±0.0798c | 1.1627±0.2409c |
| *Streptomyces* | 1.0467±0.1802b | 1.9759±0.2294a | 1.1793±0.0519b | 0.9049±0.4172b | 1.6674±0.4044ab | 1.2961±0.8118ab |
| *Neorhizobium- Rhizobium* | 1.0528±0.1278c | 1.9055±0.2506a | 1.5559±0.2076b | 0.7605±0.0362d | 1.499±0.149b | 1.281±0.0979bc |
| *Arachidicoccus* | 1.4689±0.3178a | 1.4417±0.3307a | 1.3402±0.0583a | 0.9299±0.0474b | 0.6707±0.1106b | 0.6949±0.0779b |
| *Bryobacter* | 1.4622±0.1069a | 0.5826±0.0664c | 0.7678±0.0526bc | 1.5639±0.2196a | 0.9341±0.2022b | 0.9069±0.1123b |
| *Paenarthrobacter* | 0.2664±0.1112c | 1.7934±0.4009a | 1.0345±0.0763b | 0.2851±0.1316c | 1.5748±0.1145a | 1.2164±0.2979b |
| *Rhodanobacter* | 1.3208±0.076a | 1.4215±0.1648a | 0.6036±0.0786b | 0.6712±0.0769b | 0.6922±0.0189b | 0.712±0.5405b |
| *Flavobacterium* | 0.8198±0.3053ab | 0.8002±0.149ab | 0.9403±0.2141ab | 0.907±0.2327ab | 0.5902±0.0912b | 1.0675±0.1418a |
| *Nocardioides* | 0.7048±0.0802a | 0.6967±0.0842a | 0.8963±0.0309a | 0.738±0.1751a | 0.8383±0.0401a | 0.9193±0.2548a |
| *Luteimonas* | 0.5715±0.1255c | 0.8554±0.2048b | 0.8651±0.0949b | 0.6774±0.0952bc | 0.5849±0.1231c | 1.132±0.0656a |
| *Nitrospira* | 0.8039±0.0673b | 0.2555±0.0278d | 0.511±0.0525c | 1.2175±0.1087a | 0.6607±0.1640bc | 0.6504±0.136bc |
| *Chitinophaga* | 0.5342±0.2591b | 0.9353±0.1827a | 0.3468±0.0822b | 1.0021±0.2066a | 0.3612±0.1111b | 0.5739±0.1384b |
| *Paenibacillus* | 0.2594±0.0231c | 0.9462±0.0794a | 0.9185±0.0834a | 0.2254±0.0314c | 0.852±0.1893a | 0.5347±0.0755b |
| *Candidatus_Udaeobacter* | 0.7922±0.1256a | 0.2312±0.0427c | 0.5648±0.1288b | 0.9128±0.0719a | 0.5176±0.1201b | 0.5412±0.1884b |
| *Lysobacter* | 0.2995±0.0329c | 0.5272±0.1234b | 0.9373±0.1605ab | 0.3719±0.1057c | 0.3926±0.0540bc | 0.9638±0.1268a |
| **Here;** 0 kg/ha of biochar + 105 kg/ha of pure nitrogen (B0N105), 600 kg/ha of biochar + 105 kg/ha of pure nitrogen (B600N105), 1200 kg/ha of biochar + 105 kg/ha of pure nitrogen (B1200N105), 0 kg/ha of biochar + 126 kg/ha of pure nitrogen (B0N126), 600 kg/ha of biochar + 126 kg/ha of pure nitrogen (B600N126), and 1200 kg/ha of biochar + 126 kg/ha of pure nitrogen (B1200N126).Significant differences among treatments represents by different small letters within a column according to LSD-test at *p < 0.05*. | | | | | | |
